# Molecular Mechanisms of the RECQ4 Pathogenic Mutations

**DOI:** 10.3389/fmolb.2021.791194

**Published:** 2021-11-18

**Authors:** Xiaohua Xu, Chou-Wei Chang, Min Li, Chao Liu, Yilun Liu

**Affiliations:** Department of Cancer Genetics and Epigenetics, Beckman Research Institute, City of Hope, Duarte, CA, United States

**Keywords:** RECQ helicase, cancer, aging, DNA replication, DNA repair, mitochondria

## Abstract

The human *RECQ4* gene encodes an ATP-dependent DNA helicase that contains a conserved superfamily II helicase domain located at the center of the polypeptide. RECQ4 is one of the five RECQ homologs in human cells, and its helicase domain is flanked by the unique amino and carboxyl termini with sequences distinct from other members of the RECQ helicases. Since the identification of the *RECQ4* gene in 1998, multiple RECQ4 mutations have been linked to the pathogenesis of three clinical diseases, which are Rothmund-Thomson syndrome, Baller-Gerold syndrome, and RAPADILINO. Patients with these diseases show various developmental abnormalities. In addition, a subset of RECQ4 mutations are associated with high cancer risks, especially for osteosarcoma and/or lymphoma at early ages. The discovery of clinically relevant *RECQ4* mutations leads to intriguing questions: how is the RECQ4 helicase responsible for preventing multiple clinical syndromes? What are the mechanisms by which the RECQ4 disease mutations cause tissue abnormalities and drive cancer formation? Furthermore, RECQ4 is highly overexpressed in many cancer types, raising the question whether RECQ4 acts not only as a tumor suppressor but also an oncogene that can be a potential new therapeutic target. Defining the molecular dysfunctions of different RECQ4 disease mutations is imperative to improving our understanding of the complexity of RECQ4 clinical phenotypes and the dynamic roles of RECQ4 in cancer development and prevention. We will review recent progress in examining the molecular and biochemical properties of the different domains of the RECQ4 protein. We will shed light on how the dynamic roles of RECQ4 in human cells may contribute to the complexity of RECQ4 clinical phenotypes.

## Introduction

The *RECQ4* gene was first described in 1998 as one of the two last members of the RECQ helicase family identified based on their shared homology in the superfamily II (SFII) helicase domain (Kitao et al., 1998). Subsequently, mutations in the *RECQ4* gene have been linked to the pathogenesis of three clinical diseases, which are Rothmund-Thomson syndrome (RTS), Baller-Gerold syndrome (BGS), and RAdial ray malformations, PAtellae hypo/aplasia and cleft or highly arched palate, DIarrhea and dislocated joints, LIttle size and limb malformation, NOse slender and normal intelligence (RAPADILINO) (Kitao et al., 1999a; Larizza et al., 2006; Siitonen et al., 2009). Premature aging, skeletal abnormalities, juvenile cataracts, skin hyperpigmentation and widened blood capillaries known as poikiloderma are the common clinical features associated with RTS. Immunodeficiency has also been reported in patients suffering RTS (De Somer et al., 2010; Smeets et al., 2014). RTS-associated abnormalities in bone development were recapitulated in the mouse models (Lu et al., 2015; Ng et al., 2015; Castillo-Tandazo et al., 2021). Some of the clinical features observed in the RTS patients are also common in the BGS patients (Mégarbané et al., 2000). Nonetheless, RTS and BGS remain as two separate syndromes, since BGS also displays craniosynostosis during embryonic development, which is not shared with RTS. Similarly, RAPADILINO patients also suffer clinical features partially overlapping with the RTS patients. Through these clinical overlaps, scientists were able to link mutations in the *RECQ4* gene to RAPADILINO (Siitonen et al., 2003).

To date, over 100 of clinically relevant mutations have been identified throughout the *RECQ4* gene (Table 1). In addition to developmental abnormalities and premature aging, cancer predisposition is characteristic of the human diseases linked to several *RECQ4* mutations (Liu, 2010). Defining the functions and regulation of human RECQ4 is critical for advancing our knowledge of the fundamental biology of development, aging, and cancer. This review will provide an overview on the efforts made during the past 2 decades to dissect the biochemistry, cellular functions, and regulations of the RECQ4 protein. We will discuss how the biochemical and cellular properties of RECQ4 may be affected by the clinical mutations.

**TABLE 1:** RECQ4 mutations with clinical implications. (del) deletion; (>) nucleotide change from; (X) premature stop codon; (dup) duplication; (fs) frameshift; (ins) insertion.

Mutation	Effect	Mutation location	Syndrome	Cancer type	References
c.84+6del16	Missplicing	SLD2, MTS	RTS	—	Siitonen et al. (2009)
c.118 + 27del25	Missplicing	SLD2, MTS	RTS	—	Broom et al. (2006)
c.119-1G > A	Missplicing	SLD2, MTS	RTS	—	Zhang et al. (2021)
c. 160_161insGGGCC	p.Gln54X	SLD2, MTS, NTS	RTS	—	Wang et al. (2018)
c.161A > G	p.Gln54Arg	SLD2, MTS, NTS	RTS	—	Suter et al. (2016)
c.212A > G	p.Glu71Gly	SLD2, MTS	RTS	—	Suter et al. (2016)
c.226delC	p.Arg76X	SLD2, MTS	—	Metachronous gastric cancer	Sakuta et al. (2020)
c.358G > A	p.Gly120Arg	SLD2	—	Esophageal squamous cell cancer	Zeng et al. (2021)
c.496C > T	p.Gln166X	SLD2	BGS	—	Siitonen et al. (2009)
c.558_564dup	p.Gly189fsX	SLD2	RTS	—	Suter et al. (2016)
c.691G > A	p.Gly231Ser	N-terminus	RTS	—	Gui et al. (2018)
c.806G > A	p.Trp269X	N-terminus	RAPADILINO	Lymphoma	Siitonen et al. (2003)
c.853_854del	p.Pro285X	N-terminus	—	Metachronous gastric cancer	Sakuta et al. (2020)
c.866C > G	p.Ala289Gly	N-terminus	—	Metachronous gastric cancer	Sakuta et al. (2020)
c.910C > T	p.Gln304X	N-terminus	RTS	—	Wang et al. (2018)
c.978_979delTCinsG	p.Ser326fsX	N-terminus	RTS	—	Yadav et al. (2019)
c.1048_1049delAG	p.Arg350fsX	N-terminus	RTS	Lymphoma	Wang et al. (2003), Suter et al. (2016), van Rij et al. (2017), Colombo et al. (2018)
c.1078C > T	p.Gln360X	N-terminus	RTS	Squamous cell carcinoma, basal cell carcinoma	Suter et al. (2016)
c.1132–2A > G	missplicing	N-terminus	RTS	—	Yadav et al. (2019)
c.1222C > T	p.Gln408X	ZnK	RTS	—	Suter et al. (2016)
c.1390+2delT	p.Ala420_Ala463del	NTS, MTE, ZnK	RAPADILINO	Lymphoma	Siitonen et al. (2003)
c.1391-1G > A	Missplicing	NTS, MTE	RTS	Osteosarcoma	Lindor et al. (2000), van Rij et al. (2017)
c.1397C > T	p.Pro466Leu	NTS, MTE	RAPADILINO	—	Siitonen et al. (2009)
c.1483 + 25del11	Missplicing	SF2	RTS	Osteosarcoma	(Wang et al., 2002; Wang et al., 2003)
c.1483 + 27del11	Missplicing	SF2	RTS	Osteosarcoma	Balraj et al. (2002)
c.1568G > C_1573delT	Missplicing	SF2	RTS	—	Colombo et al. (2018)
c.1568delG	p.Ser523fsX	SF2	RTS	—	Cabral et al. (2008)
c.1573delT	p.Cys525fsX	SF2	RTS, BGS, RAPADILINO	Osteosarcoma, breast cancer	Van Maldergem et al. (1992), Lindor et al. (1996), Lindor et al. (2000), Pujol et al. (2000), Beghini et al. (2003), Wang et al. (2003), Kellermayer et al. (2005), Van Maldergem et al. (2006), Salih et al. (2018), Shahi et al. (2019)
c.1580C > T	p.Thr527Met	SF2	—	Esophageal squamous cell cancer	Zeng et al. (2021)
c.1649C > G	p.Ala550Gly	SF2	—	esophageal squamous cell cancer	Zeng et al. (2021)
c.1650del7	p.Ala551fsX	SF2	RTS	Osteosarcoma	(Lindor et al., 1996; Kitao et al., 1999a)
c.1697T > C	p.Leu566Pro	SF2	RTS	—	Suter et al. (2016)
c.1704G > A	Missplicing	SF2	RTS	—	Wang et al. (2003)
c.1704+1G > A	Missplicing	SF2	RTS	Lymphoma	Simon et al. (2010)
c.1705-1G > A	Missplicing	SF2	RTS	—	Sznajer et al. (2008)
c.1718delA	p.Gln573fsX	SF2	RTS	Osteosarcoma	Wang et al. (2003)
c.1724_1725delAC	p.His575fsX	SF2	RTS	—	Gui et al. (2018)
c.1763delG	p.Gly588fsX	SF2	—	Metachronous gastric cancer	Sakuta et al. (2020)
c.1770_1807del	p.Pro591fsX	SF2	RTS	—	Gui et al. (2018)
c.1878+5G > A	Missplicing	SF2	RTS	—	Wang et al. (2003)
c.1878 + 32_1879-27del24	Missplicing	SF2	RTS	Osteosarcoma	(Wang et al., 2003; Gutiérrez-Jimeno et al., 2020)
c.1878 + 32_1878 + 55del	missplicing	SF2	RTS	—	Colombo et al. (2018)
c.1885del4	p.Arg629fsX	SF2	RAPADILINO	—	Siitonen et al. (2009)
c.1887del4	p.Glu630fsX	SF2	RAPADILINO	—	Siitonen et al. (2009)
c.1892G > T	p.Arg631Leu	SF2	—	Esophageal squamous cell cancer	Zeng et al. (2021)
c.1910T > C	p.Phe637Ser	SF2	RAPADILINO	—	Siitonen et al. (2009)
c.1913T > C	p.Leu638Pro	SF2	RTS	Lymphoma	Sznajer et al. (2008), Siitonen et al. (2009)
c.1919_1924delTCACAG	p.Leu640_Ala642delinsP	SF2	RTS	Lymphoma	Simon et al. (2010)
c.1930_1935dup	p.Ala644_Thr645 dup	SF2	RTS	—	Suter et al. (2016)
c.2059-1G > C	missplicing	SF2	BGS	—	Cao et al. (2015)
c.2085delA	p.Leu695fsX	SF2	RTS	—	Suter et al. (2016)
c.2091T > G	p.Phe697Leu	SF2	RAPADILINO	—	Siitonen et al. (2009)
c.2141_2142delAG	p.Glu714fsX	SF2	BGS	—	Cao et al. (2015)
c.2149G > T	p.Ala717Ser	SF2	—	Esophageal squamous cell cancer	Zeng et al. (2021)
c.2207ins1	p.Lys738fsX	SF2	RTS	—	Wang et al. (2003)
c.2269C > T	p.Gln757X	SF2	RTS	Osteosarcoma	Lindor et al. (1996), Kitao et al. (1999b), Pujol et al. (2000), Cabral et al. (2008), Salih et al. (2018), Gutiérrez-Jimeno et al. (2020), Zhang et al. (2020)
c.2272C > T	p.Arg758X	SF2	RTS	Esophageal squamous cell cancer	Colombo et al. (2014), Zeng et al. (2021)
c.2290C > T	p.Gln764X	SF2	RTS	—	Zhang et al. (2021)
—	p.Arg766GlyfsX	SF2	—	Ampullary carcinoma, lung adenocarcinoma, T cell lymphoma, chondroblastic osteosarcoma, renal cell carcinoma, neuroblastoma, hepatoblastoma, B-lymphoblastic leukemia/lymphoma, acute myeloid leukemia, optic nerve glioma	cBioPortal
c.2335del22	p.Asn779fsX	SF2	BGS	—	Siitonen et al. (2009)
c.2398C > T	p.Gln800X	SF2	RTS	—	Siitonen et al. (2009)
c.2419ins5	p.Arg807fsX	SF2	RTS	Lymphoma	Siitonen et al. (2009)
c.2421dupT	p.Asp808fsX	SF2	RTS	—	Gui et al. (2018)
c.2428C > T	p.Gln810Cys	SF2	RTS	—	Wang et al. (2003)
c.2461C > T	p.Gln821X	C-terminus	RTS	—	Siitonen et al. (2009)
c.2464-1G > C	Missplicing	C-terminus	RTS	—	Wang et al. (2003), Siitonen et al. (2009)
c.2476C > T	p.Arg826X	C-terminus	RTS, RAPADILINO	—	Wang et al. (2003), Siitonen et al. (2009)
c.2492_2493delAT	p.His831fsX	C-terminus	RTS, BGS	Osteosarcoma, lymphoma	Debeljak et al, (2009), Colombo et al. (2014), Gutiérrez-Jimeno et al. (2020)
c.2506_2518del13	p.Trp836fsX	R4ZBD	BGS	Lymphoma	Debeljak et al. (2009)
c.2547−2548delGT	p.Phe850fsX	R4ZBD	RTS	Osteosarcoma	Wang et al. (2003)
c.2552delC	p.Pro851GlnfsX	R4ZBD	RTS	—	Wang et al. (2003)
c.2569_2574dup	p.Cys857_T858 dup	R4ZBD	RTS	—	Suter et al. (2016)
c.2636del	p.Pro879X	R4ZBD	—	Prostate cancer	Paulo et al. (2018)
c.2752G > T	p.Glu918X	R4ZBD, NES	RTS	—	Wang et al. (2018)
c.2767_2768delTT	p.Leu923fsX	R4ZBD	RTS, RAPADILINO	—	Fradin et al. (2013)
c.2780T > G	p.Leu927Arg	R4ZBD	RTS	—	Cabral et al. (2008)
c.2789_2812del	p.His930_Leu937del	R4ZBD	RTS	—	Suter et al. (2016)
c.2886-1G > A	Missplicing	R4ZBD	RTS	—	Zhang et al. (2021)
c.2886-2A > T	Missplicing	R4ZBD	RTS	—	Broom et al. (2006)
c.3014delG	p.Arg1005fsX	R4ZBD	—	Metachronous gastric cancer	Sakuta et al. (2020)
c.3014_3015AG	p.Arg1005Gln	R4ZBD	—	Metachronous gastric cancer	Sakuta et al. (2020)
c.3016delG	p.Ala1006fsX	R4ZBD	—	Metachronous gastric cancer	Sakuta et al. (2020)
c.3021_3022del	p.Cys1008fsX	R4ZBD	RTS	—	Colombo et al. (2018)
c.3056-2A > C	Missplicing	R4ZBD	BGS	—	Van Maldergem et al. (2006)
c.3061C > T	p.Arg1021Trp	R4ZBD	RTS, BGS, RAPADILINO	—	Fradin et al. (2013), Wang et al. (2018)
c.3062G > A	p.Arg1021Gln	R4ZBD	RTS	—	Wang et al. (2018)
c.3072_3073delAG	p.Val1026fsX6	R4ZBD	RTS	Osteosarcoma	Wang et al. (2003)
c.3072delA	p.Val1026fsX18	R4ZBD	RAPADILINO	—	Siitonen et al. (2009)
c.3124_3127ACC	Missplicing	R4ZBD	—	Metachronous gastric cancer	Sakuta et al. (2020)
c.3151A > G	p.Ile1051Val	C-terminus	BGS	—	Siitonen et al. (2009)
c.3214A > T	p.Arg1072X	C-terminus	RAPADILINO	—	Siitonen et al. (2003)
c.3236G > T	p.Ser1079Ile	C-terminus	RTS	—	Colombo et al. (2018)
c.3270delG	p.Glu1090fsX	C-terminus	RTS	—	Wang et al. (2003)
c.3271C > T	p.Gln1091X	C-terminus	RAPADILINO	—	Siitonen et al. (2003)
c.3276delG	p.Asp1093fsX	C-terminus	RTS	Osteosarcoma	Wang et al. (2003)
c.3330dupA	pGlu1111fsX	Acidic patch	RTS	—	Suter et al. (2016)
c.3409G > A	p.Asp1137Asn	C-terminus	—	Metachronous gastric cancer	Sakuta et al. (2020)
c.3501-3502delCG	Missplicing	C-terminus	RTS	—	Wang et al. (2018)
c.3523C > T	p.Gln1175X	NES	RTS	—	Wang et al. (2003)
c.3552dupG	p.Arg1185fsX	NES	RTS	—	Zhang et al. (2021)
c.3573C > G	p.Ser1191Arg	C-terminus	RTS	—	Suter et al. (2016)
c.3599_3600delCG	p.Thr1200fsX	C-terminus	RAPADILINO	—	Siitonen et al. (2009)

## RECQ4 in DNA Replication Initiation

**FIGURE 1:**
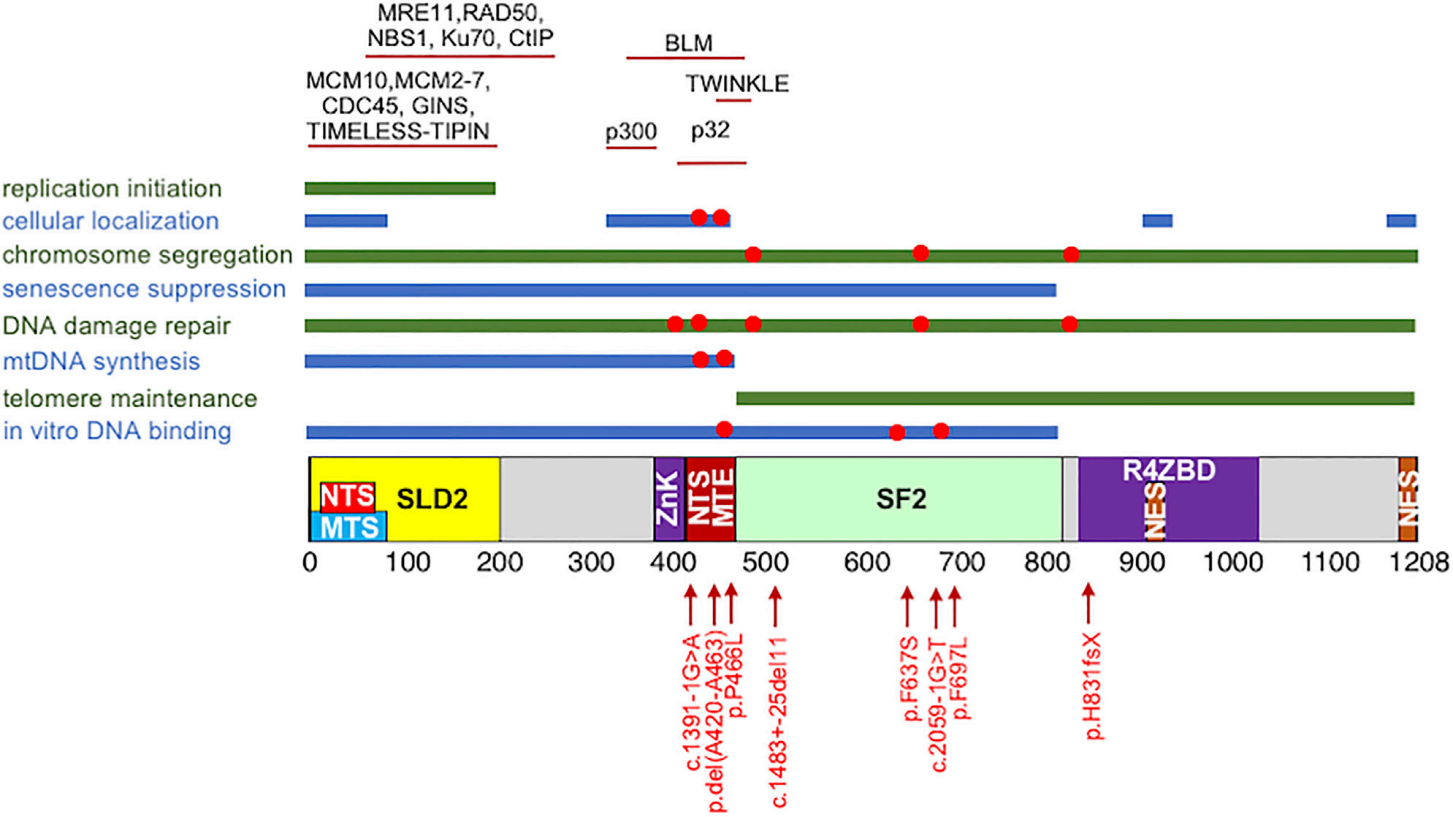
Schematic of the human RECQ4 protein domains, including the SLD2 (yellow) and conserved SF2 helicase domains (green). NTS: nuclear targeting signal. MTS: mitochondrial targeting signal. ZnK: zinc knuckle. MTE: mitochondrial exclusion; NES: nuclear export signal. R4ZBD: RECQ4 zinc binding domain. **(Top)** Protein-protein interactions domains. **(Middle)** RECQ4 cellular functions and the domains involved. **(Bottom)** The disease-associated mutations that have been implicated in the RECQ4 cellular functions, as indicated with red circles in the middle.

Human RECQ4 is a 1,208 amino-acid (a.a.) long protein. In addition to the SFII helicase domain between residues 470 and 820 located at the center of the polypeptide (Figure 1), RECQ4 also contains unique amino (N) and carboxyl (C) termini that are not shared by other members of the RECQ family in their sequences (Kitao et al., 1998; Liu, 2010; Ellis et al., 1995; Yu et al., 1996). Two mouse models highlight the essential role of the RECQ4 N-terminus in embryonic development. The first attempt to generate viable RECQ4 knockout mice was not successful, as the mice died at the embryonic stage between day 3.5 and 6.5 (Ickikawa et al., 2002). However, mice expressing intact N-terminal fragment survived (Mann et al., 2005). Similarly, chicken DT40 cells lacking full-length RECQ4 underwent apoptosis but were rescued by expressing the N-terminal fragment containing the first 496 residues (Abe et al., 2011). The molecular function of the RECQ4 N-terminus was uncovered when a yeast Sld2-like domain was identified within the first 200 a. a. of the RECQ4 protein (Figure 1) (Sangrithi et al., 2005; Matsuno et al., 2006). Consistent with the critical role of yeast Sld2 in DNA replication, it was first demonstrated that *xenopus* RECQ4 also functions in DNA replication initiation (Sangrithi et al., 2005; Matsuno et al., 2006). The involvement of RECQ4 in DNA replication initiation is also conserved in human cells (Im et al., 2009; Xu et al., 2009; Thangavel et al., 2010). The N-terminus of RECQ4 alone is sufficient in recruiting essential replication factors to the origin of replication to initiate DNA synthesis (Shin et al., 2019). Through the SLD2 domain, human RECQ4 forms cell cycle-dependent, chromatin-bound protein complexes containing core replisome factors MCM10, MCM2-7 helicase, CDC45 and GINS at replication origins (Xu et al., 2009). Additional studies revealed that the activation of MCM2-7 replicative helicase activity through the formation of a stable CDC45-MCM2-7-GINS (CMG) complex requires RECQ4 and MCM10 (Im et al., 2009). Furthermore, the formation and retention of the RECQ4-CMG complex on chromatin is restricted within the S-phase of the cell cycle by checkpoint protein TIMELESS (Xu et al., 2016). Given the important role of RECQ4 in DNA replication initiation, it is perhaps not a surprise that the association of RECQ4 and MCM10 with replication origins is subjected to negative regulation by the DNA damage checkpoint control to suppress S-phase entry in response to DNA damage (Im et al., 2015).

The dependency of cell growth on the RECQ4 SLD2 domain is expected to impose a selection pressure against mutations within this domain in the human population, as changes that abolish RECQ4 function in DNA replication initiation may be lethal. Replication defect may also explain why mutations in the RECQ4 N-terminus trigger replicative senescence (Lu et al., 2014). Moreover, additional functions of the N-terminal RECQ4 may be required for cell survival. For example, the N-terminus of RECQ4 is required for proper chromosome segregation to prevent G_2_/M cell cycle arrest and mitotic cell death (Fang et al., 2018). RECQ4 may promote proper chromosome segregation by stabilizing Aurora B Kinase important for mitotic spindle assembly through a direct protein-protein interaction or via its association with the microtubules to ensure correct chromosome alignment (Yokoyama et al., 2019). Nonetheless, in chicken DT40 cells, even though RECQ4-MCM10 interaction is important for efficient replication origin firing, defects in this interaction do not lead to cell death (Kliszczak et al., 2015). In addition, a significant number of the disease associated mutations have been found within the RECQ4 SLD2 motif (Table 1). It is possible that these mutations do not impair DNA replication or chromosome segregation. Alternatively, additional pathway(s) to mediate DNA replication initiation or chromosome segregation exists in chicken DT40 cells or is activated in patient cells to support cell viability.

## RECQ4 in Mitochondrial Biogenesis

RECQ4 not only localizes in the nucleus to initiate DNA replication but is also present in the cytoplasm (Ding and Liu, 2015; Croteau et al., 2012a; Wang et al., 2014; De et al., 2012). RECQ4 contains two nuclear targeting signals (NTS; Figure 1), one of which overlaps with residues missing in the highly cancer prone RECQ4 del(Ala420-Ala463) mutant protein associated with RAPADILINO and is important in negatively regulating RECQ4 cytoplasmic localization (Burks et al., 2007). As a consequence, the cancer mutant protein missing residues between Ala420 and Ala463 is cytoplasmic (Burks et al., 2007). Several lysine residues located immediately upstream of Ala420-Ala463 residues are modified by the p300 acetyltransferase, and their acetylation also promotes cytoplasmic localization (Dietschy et al., 2009). Further studies revealed that the increasing amount of the cytoplasmic del(Ala420-Ala463) mutant proteins is partly due to their accumulation in the mitochondria (Wang et al., 2014). Specifically, the missing residues in the del(Ala420-Ala463) mutant are involved in binding to hyaluronan binding protein HABP1/p32, which acts as a negative regulator of RECQ4 mitochondrial localization (Wang et al., 2014). Hence, the residues between Ala420 and Ala463 also serve as a mitochondrial exclusion (MTE) signal. Interestingly, while missing NTS/MTE enhances RECQ4 mitochondrial localization (Wang et al., 2014), deletion of two nuclear export signals (NES; Figure 1) found near the C-terminal end of the protein reduces mitochondrial localization (Chi et al., 2012). In addition, the first 84 residues of the RECQ4 protein contain a mitochondrial targeting signal (MTS; Figure 1) that also plays a positive role in targeting RECQ4 mitochondrial localization (De et al., 2012). It is clear that RECQ4 contains multiple regulatory motifs to balance the levels of RECQ4 in the nucleus and mitochondria.

Studies using RECQ4 disease mutations reveal a role of RECQ4 in mitochondrial biogenesis. For example, RECQ4 is involved in mitochondrial DNA (mtDNA) synthesis and maintenance, and abnormal mtDNA levels were observed in the RECQ4 mutant cells (Croteau et al., 2012a; De et al., 2012; Wang et al., 2014; Chang et al., 2020). The changes in mtDNA copy numbers and their contents correlate with the mitochondrial dysfunction phenotypes in human cells (Croteau et al., 2012a; De et al., 2012; Wang et al., 2014; Chang et al., 2020). RECQ4 interacts with mitochondrial replication factors, including TWINKLE/PEO1, TFAM and DNA polymerase γ (Croteau et al., 2012a; Wang et al., 2014). The interaction of RECQ4 with TWINKLE is enhanced by the del(Ala420-Ala463) mutation due to a decrease in the interaction with the negative regulator p32, resulting in RECQ4 mitochondrial accumulation and elevated mtDNA synthesis rate to increase mtDNA copy number (Wang et al., 2014). As a consequence, cells expressing RECQ4 del(Ala420-Ala463) mutant protein show altered oxidative phosphorylation efficiency for mitochondrial-dependent ATP production (Croteau et al., 2012a; Wang et al., 2014; Kumari et al., 2016). Interestingly, an adjacent RAPADILINO mutation Pro466Leu also impairs RECQ4 interaction with p32, leading to the accumulation of the mutant protein in the mitochondria (Chang et al., 2020). However, unlike cells expressing the del(Ala420-Ala463) mutant proteins, Pro466Leu expression does not accelerate mtDNA synthesis despite the fact that the point mutation also enhances RECQ4-TWINKLE interaction (Chang et al., 2020). These observations highlight the heterogeneity in mitochondrial dysfunction observed in different RECQ4 patient cell lines (Croteau et al., 2012a). RECQ4 also interacts with and promotes the transport of p53 to mitochondria for mtDNA maintenance, and this interaction is disrupted in response to stress to allow the recruitment of p53 to the nucleus (De et al., 2012). The functional interaction between RECQ4 and p53 is further supported by the overlapping mtDNA mutations found between cells carrying the RTS-associated RECQ4 mutations and those cells isolated from Li-Fraumeni syndrome patients containing p53 mutations (Gupta et al., 2014). RECQ4 deficiency also contributes to increasing autophagy likely due to mitophagy impairment (Duan and Fang, 2016).

## RECQ4 in DNA Repair

In addition to participating in DNA synthesis in both the nucleus and the mitochondria, studies also reported that RECQ4 participates in DNA damage response and repair. For example, RECQ4 is recruited to DNA damage sites, and this re-localization requires residues between 363 and 492 (Singh et al., 2010). RECQ4 has been implicated in non-homologous end joining via its N-terminal interaction with Ku70 during G_1_ phase (Figure 1) (Lu et al., 2017; Shamanna et al., 2014). During S/G_2_ phase, CDK-mediated phosphorylation of the RECQ4 N-terminus at the S89 and S251 residues and DDB1-CUL4A mediated ubiquitination after ionizing radiation switch RECQ4 interaction in favor of MRE11 for homologous recombination (Lu et al., 2017). RECQ4 N-terminus also binds to CtIP to recruit MRE11-RAD50-NBS1 complex to the DNA break site to facilitate homologous recombination (Figure 1) (Lu et al., 2016). Residues between 363 and 492 important for the RECQ4 recruitment to DNA damage sites also interact with Bloom’s syndrome helicase BLM (Figure 1), a RECQ homolog, in a DNA damage-dependent manner (Singh et al., 2012). BLM is not required for the recruitment but for the retention of RECQ4 at the DNA damage site (Singh et al., 2010; Singh et al., 2012). RECQ4 interaction also stimulates BLM helicase activity *in vitro* (Singh et al., 2012). However, the extent to which the two helicases function in the same repair pathway remain to be determined, as different sensitivities to DNA damaging agents have been observed between pre-B lymphocyte cells depleted with RECQ4 and BLM (Kohzaki et al., 2012). Both BLM-deficient mice and RECQ4 conditional mutant mice exhibit bone marrow failure, but the hematopoietic defects can be rescued by p53 deletion only in the BLM-deficient mice but not in RECQ4-deficient mice (Smeets et al., 2014). Given that RECQL4 depletion has additive effects on proliferation and sister chromatin exchange in BLM-deficient cells, these two RECQ proteins may function in non-overlapping pathways in DNA damage response and repair (Singh et al., 2012). Analysis of the point mutations in either the *BLM* or *RECQ4* genes that specifically abolishes the interaction between these two proteins may provide insights into the function of this interaction.

In addition to the N-terminus, the conserved SF2 and the C-terminal domains are also central to the regulation of DNA damage response and repair. Cells isolated from a RTS patient compound heterozygous of RECQ4 mutations missing the SF2 and the C-terminus of RECQ4 showed defects in ATM activation after DNA damage (Park et al., 2019). In addition, RECQ4 depleted cells complemented with only the N-terminal fragment were viable, but these cells exhibited increasing sensitivity to DNA damaging agents (Abe et al., 2011; Kohzaki et al., 2012). Even though the residues required for RECQ4 recruitment to DNA damage sites have been identified (Singh et al., 2010), it is yet to be determined if these residues are involved in direct DNA damage recognition or RECQ4 re-localization to the damage site via protein-protein interaction(s). Once bound to the DNA damage, RECQ4 requires RNF8-dependent ubiquitination at its C-terminus to dissociate from the site of DNA damage, allowing downstream DNA repair factors to bind to the damage site (Tan et al., 2021). While studies show that RECQ4 positively regulates homologous recombination and non-homologous end joining, RECQ4 suppresses the repair of DNA breaks *via* RAD52-mediated single-strand annealing pathway via its SF2 and/or the C-terminal domains (Kohzaki et al., 2020). Upon oxidative stress, a fraction of the RECQ4 proteins re-localize to the nucleolus, and the nucleolar localization is dependent on PARP1, which poly(ADP-ribosyl)ates the C-terminus of RECQ4 *in vitro* (Woo et al., 2006). RECQ4 may also participate in base excision repair in response to oxidative stress (Schurman et al., 2009).

## RECQ4 Biochemical Properties

*In vitro*, the N-terminus of RECQ4 binds to various DNA substrates, including double-stranded, single-stranded and splayed-arm DNA (Xu and Liu, 2009; Keller et al., 2014). Multiple regions within the N-terminal fragment participate in nucleic acid binding. For example, the first 54 residues of the RECQ4 protein exhibit affinity toward DNA (Ohlenschläger et al., 2012). RECQ4 also contains a conserved zinc knuckle motif between residues 397 and 421 that preferentially binds to RNA over DNA (Marino et al., 2016). Structural analysis located a second RECQ4 zinc-binding domain (R4ZBD; Figure 1) at the C-terminus of RECQ4 between residues 836 and 1,044, but R4ZBD does not play a significant role in DNA binding or ATPase activities (Kaiser et al., 2017). The nucleic acid binding by the N-terminal fragment of RECQ4 contributes to its strand annealing and strand exchange activities (Xu and Liu, 2009; Keller et al., 2014), providing an explanation for why pathogenic mutations within the RECQ4 N-terminus affect its annealing and strand exchange activities (Chang et al., 2020). RECQ4 N-terminus also shows high affinities toward unusual DNA structures, such as guanine quadruplex and RNA:DNA hybrids (Keller et al., 2014; Chang et al., 2020), supporting additional roles of RECQ4 N-terminus in nucleic acid metabolism, which is altered by the pathogenic RECQ4 Pro466Leu mutation. Specifically, Pro466Leu mutation increases RECQ4 affinities to both DNA and RNA *in vitro* and in mitochondria, and this enhanced affinity correlates with the elevated strand annealing activity between DNA and RNA (Chang et al., 2020). As a consequence, Pro466Leu mutant cells accumulate RNA:DNA hybrids on the mtDNA that block the completion of mtDNA synthesis (Chang et al., 2020). These enhanced activities were not observed in cells expressing the del(Ala420-Ala463) mutant protein, providing an explanation for why cells expressing Pro466Leu RECQ4 mutant do not contain high levels of mtDNA compared to those expressing the del(Ala420-Ala463) mutant (Croteau et al., 2012b; Wang et al., 2014; Chang et al., 2020). It would be a great interest to determine if the enhanced nucleic acid affinity by the Pro466Leu mutation may retain RECQ4 at DNA damage sites, hindering the binding and access of downstream DNA repair factors (Tan et al., 2021). Nonetheless, another report showed that Pro466Leu, Phe637Ser and Phe697Leu disease mutations decrease RECQ4 DNA binding (Jensen et al., 2012).

In addition to strand annealing and strand exchange, both of which are intrinsic activities of the RECQ4 N-terminal fragment, the conserved SF2 domain contain active ATPase and helicase activities (Rossi et al., 2010; Suzuki et al., 2009). Surprisingly, the last 92 amino acids outside of the SF2 domain are required for efficient RECQ4 helicase activity, but the mechanism for this enhancement remains unclear (Kaiser et al., 2017). RECQ4 helicase activity shows preferences toward telomeric substrates containing DNA lesions that may block DNA replication (Ferrarelli et al., 2013). In support of a role of RECQ4 at the telomere, deletion of the RECQ4 SF2 and the C-terminal domains correlates with fragile telomeres, which may be due to defects in resolving telomeric D-loop structures via the SF2 domain (Ghosh et al., 2012). Furthermore, in cells, a point mutation in either the Walker A or Walker B motif of the RECQ4 SF2 domain is sufficient to abolish ATM activation, suggesting that the helicase activity is required for this function (Park et al., 2019). On the other hand, ATPase and helicase activities are not required for RECQ4 recruitment to DNA damage sites (Jensen et al., 2012), arguing that RECQ4 recruitment to the DNA damage site is independent of the ATM activation. It is possible that the helicase activity plays a role in DNA end resection to promote homologous recombination at the DNA break site (Lu et al., 2016). Similar to humans (Table 1), mice carrying a SF2 truncation mutation suffered RTS phenotypes including aneuploidy and increasing cancer incidents and bone marrow failure, suggesting that the RECQ4 helicase activity is required for disease prevention (Mann et al., 2005; Castillo-Tandazo et al., 2019). However, in contrast to the observations reported using cell culture and in mice carrying the SF2 truncation mutation mentioned above, mice with only a Walker A point mutation to abolish the ATPase and helicase activities went through normal development and showed no defects in hematopoiesis or increasing sensitivity to DNA damage agents (Castillo-Tandazo et al., 2019). Most likely, other biochemical properties of the RECQ4 SF2 domain independent of the helicase activity are important for disease prevention and normal development.

RECQ4 is a highly interactive protein in cells, and protein-protein interactions impact RECQ4 catalytic activities. For example, ribosomal protein S3 binds to the first 320 amino acids of RECQ4 and this interaction is enhanced by oxidative stress (Patil and Hsieh, 2017). However, this interaction inhibits RECQ4 DNA binding and helicase activities (Patil and Hsieh, 2017). Similarly, MCM10 interaction with RECQ4 promotes DNA replication initiation, but the association with MCM10 also suppresses RECQ4 strand exchange activity *in vitro* (Xu et al., 2009). Interestingly, RECQ4 N-terminus promotes mitochondrial DNA polymerase γ binding to mtDNA (Gupta et al., 2014), but the interaction with DNA polymerase γ negatively regulates RECQ4 helicase activity (Croteau et al., 2012a). The functional significance of these inhibitions on RECQ4 catalytic activities via protein-protein interactions remains unclear.

## Discussion

Extensive literature provides insights into the molecular functions of RECQ4 in DNA replication, mitochondrial biogenesis, and DNA damage response and repair. These findings are critical to our understanding of the etiology of the RECQ4 disease mutations. The complex functions of RECQ4 emphasize the importance of not treating all RECQ4 patient cell lines the same. For an example, both del (Ala420_Ala463) and Pro466Leu mutations are associated with RAPADILINO. However, while del (Ala420_Ala463) mutation is associated with high lymphoma incidents, which occurred in approximately 40% of the individuals homozygous or compound heterozygous for the mutation, the adjacent Pro466Leu clinical mutation has not been linked to cancer risk (Siitonen et al., 2003; Siitonen et al., 2009). Analysis of the mutant cell lines showed that mutations in this region impact RECQ4 mitochondrial localizations in both mutant cells, but the mutations affect mtDNA synthesis efficiency differently (Chang et al., 2020). Changes in the mtDNA copy numbers have been linked to lymphomagenesis (Kopinski et al., 2021). It remains to be determined if the difference in mtDNA synthesis efficiencies contributes to high incidents of lymphoma in patients carrying del (Ala420_Ala463) mutation, but not in those with Pro466Leu mutation. Alternatively, defects in DNA repair factors contribute to lymphoma (Knittel et al., 2018), and it is possible that the failure in recruiting RECQ4 to DNA damage sites and facilitating DNA damage repair due to the del (Ala420_Ala463) mutation contributes to lymphomagenesis (Singh et al., 2010; Singh et al., 2012). If so, it is necessary to demonstrate the Pro466Leu mutant cells are proficient in RECQ4 recruitment to DNA damage sites.

In addition to the del (Ala420_Ala463) mutation at the N-terminus of RECQ4, high cancer risks have also been reported in patients carrying the C-terminal truncation mutation Q757X (Siitonen et al., 2009), highlighting the importance of multiple RECQ4 domains in cancer prevention. Interestingly, a splicing mutation that produces a C-terminal truncated RECQ4 protein (R766X) similar to that of Q757X mutation has been reported as a recurrent hotspot in the tumor registry and considered oncogenic (cBioPortal cBioPortal. Av). Expression of the Q757X mutation also reprograms fibroblast to induced pluripotent stem cells (iPSCs) that can undergo cellular differentiation (Gatinois et al., 2020). Since increasing DNA replication competency contributes to iPSC reprogramming (Mouery et al., 2020), it is reasonable to speculate that the Q757X mutation promotes iPSC reprogramming through a change in DNA synthesis efficiency. While erroneous DNA replication due to loss-of-function mutations in factors important for this process drives genomic instability and cellular transformation, increasing replication rate as a consequence of gain-of-function mutations or up-regulation of replication factors may also contribute to genomic instability and tumorigenesis via enhanced cell proliferation and replication stress (Wang et al., 18742020). It would be of great interest to determine if RECQ4 mutations such as Q757X/R766X play an oncogenic role as gain-of-function mutations in accelerating DNA synthesis to enhance cell growth and increase replication stress.

RECQ4 is overexpressed in multiple cancers, including pancreatic cancer, melanoma, prostate and ovarian cancers, and its expression is directly proportional to tumor grades (cBioPortal cBioPortal. Av; Su et al., 2010; Maire et al., 2009). Most likely, the high expression of RECQ4 is needed to support rapid cancer cell growth, further supporting a potential oncogenic role of RECQ4. Because of its high expression in cancerous cells, RECQ4 may be considered as a potential target for cancer therapy similar to other DNA replication and repair factors (Guha, 2011; Seo and Kang, 2018). Indeed, transient down regulation of RECQ4 blocks cell growth and induces PARP1-dependent apoptosis in metastatic prostate cancer cells (Su et al., 2010). In breast cancers, RECQ4 suppression not only impairs DNA synthesis but also increases cellular sensitivity to chemotherapeutic drugs possibly through reduced efficiency in DNA damage response (Arora et al., 2016). In gastric cancer cells, ectopic expression of RECQ4 also correlates with increasing resistance to DNA damage agents (Mo et al., 2016), further suggesting the potential of inhibiting RECQ4 to sensitize cells to the existing chemotherapies. Interestingly, chemoresistance due to RECQ4 overexpression may also be a consequence of deregulated transcriptional regulations. Specifically, RECQ4 was found to interact with the transcriptional factor YB1 to promote AKT-mediated phosphorylation of YB1 and YB1-dependent gene expressions including the multidrug resistance gene MDR1 (Mo et al., 2016). In addition, since the SF2 domain and the C-terminus of RECQ4 are involved in suppressing RAD52-mediated single-stranded annealing for repairing DNA breaks, cells lacking these regions of the RECQ4 protein increase sensitivity to DNA damaging agents in the presence of RAD52 inhibitors (Kohzaki et al., 2020). Therefore, identification of pharmacological inhibitors against the multifaceted roles of RECQ4 in supporting cancer cell growth and chemoresistance may provide new therapeutic strategies.

Finally, in addition to RECQ4, mutations in ANAPC1, a subunit of the anaphase promoting complex/cyclosome (APC/C), were recently identified as the second genetic risk factor for RTS (Ajeawung et al., 2019). APC/C is an ubiquitin E3 ligase with crucial roles in regulating cell cycle progression for DNA synthesis and chromosome segregation (Barford, 2020). More research is needed to examine the potential functional interactions between RECQ4 and APC/C in DNA synthesis and chromosome segregation in normal development and RTS prevention.
